# A psychologically-based taxonomy of misdirection

**DOI:** 10.3389/fpsyg.2014.01392

**Published:** 2014-12-09

**Authors:** Gustav Kuhn, Hugo A. Caffaratti, Robert Teszka, Ronald A. Rensink

**Affiliations:** ^1^Department of Psychology, Goldsmiths, University of LondonLondon, UK; ^2^Centre for Systems Neuroscience, University of LeicesterLeicester, UK; ^3^Departments of Computer Science and Psychology, University of British ColumbiaVancouver, BC, Canada

**Keywords:** misdirection, attention, magic, memory, perception, reasoning, taxonomy

## Abstract

Magicians use misdirection to prevent you from realizing the methods used to create a magical effect, thereby allowing you to experience an apparently impossible event. Magicians have acquired much knowledge about misdirection, and have suggested several taxonomies of misdirection. These describe many of the fundamental principles in misdirection, focusing on how misdirection is achieved by magicians. In this article we review the strengths and weaknesses of past taxonomies, and argue that a more natural way of making sense of misdirection is to focus on the perceptual and cognitive mechanisms involved. Our psychologically-based taxonomy has three basic categories, corresponding to the types of psychological mechanisms affected: perception, memory, and reasoning. Each of these categories is then divided into subcategories based on the mechanisms that control these effects. This new taxonomy can help organize magicians' knowledge of misdirection in a meaningful way, and facilitate the dialog between magicians and scientists.

## Introduction

Misdirection—manipulating the spectator away from the cause of a magic effect—is widely considered a central element of the practice of magic: “[m]isdirection is a principle element in the art of deception“ (Randal, 1976, p. 380), “magic is misdirection and misdirection is magic” (Hugard, 1960, p. 115), and “[m]isdirection is the meat of deception, the stuff of which illusion is made” (Leech, 1960, p. 6). But whilst many books and articles have been written on it, a clear understanding of this concept remains elusive (Lamont and Wiseman, 1999). This paper attempts to provide such an understanding. It will review previous work on this topic, attempt to determine the psychological mechanisms involved, and suggest a taxonomy based on these mechanisms, one that can help guide when and where misdirection might be best employed.

Several taxonomies of misdirection have been suggested previously; these are useful for identifying and describing many of the fundamental principles involved. Most of these taxonomies have focused on the particular ways that misdirection can be achieved. In contrast, we propose that a more natural, less arbitrary way of making sense of misdirection is by emphasizing as much as possible the underlying psychological mechanisms. In order to get a better sense of which mechanisms these might be, we will first attempt to define misdirection more precisely^1^.

## What is misdirection?

Misdirection is sometimes defined “as the intentional deflection of attention for the purpose of disguise” (Sharpe, 1988, p. 47); as such, it would encompass anything that prevents you from noticing the secret method (i.e., the technique used to bring about the observed effect). It has also been suggested that misdirection is not simply about directing attention away from the cause of a magic effect, but toward something interesting, which again prevents the spectator from noticing the method (Wonder, 1994).

Whilst some misdirection principles involve manipulating what people attend to (and thus, what they see), “real misdirection deceives not only the eye of the spectator, but his mind as well” (Leech, 1960, p. 6), More precisely, successful misdirection might manipulate not only people's perceptions, but their memory for what happened, or their reasoning about how the effect was done. A distraction that prevents people from experiencing an effect—whether by manipulating perception, memory, or reasoning—is clearly futile (Lamont and Wiseman, 1999). Misdirection is also ineffective if it allows people to see (or work out) the method, since a key aspect of magic is the witnessing of an event that is apparently impossible. If people become aware of the misdirection, the impossible becomes possible, and the magic disappears (Pareras, 2011).

Another important feature of misdirection is that the principles used should be counterintuitive. For example, attentional misdirection is particularly effective when it exploits our incorrect assumptions about perception. Phenomena such as change blindness and inattentional blindness strongly suggest that instead of being dense and complete, our visual representations are relatively sparse, with attention being the critical element in visual awareness (Rensink, 2002, 2013). Our surprise at violations of these assumptions illustrates the gap between what we believe about our perceptual systems and their actual operation (Levin et al., 2000), making it a perfect phenomenon for magicians to exploit.

Whilst central to magic, misdirection is also used in many other domains. Politicians are often accused of misdirecting the attention of the public away from bad news, and military generals occasionally use misdirection (e.g., feints) to gain advantage over their enemies (Freudenburg and Alario, 2007). Although misdirection is not used in these examples to create a magical effect, many of the principles are the same, e.g., making sure that there is no awareness of the misdirection itself (Bond and Robinson, 1988).

## Why do we need a taxonomy?

Over the years, magicians have acquired vast amounts of useful knowledge about effective misdirection. Although much of this knowledge has been discussed in theoretical articles and books, it tends to be described only in the context of individual magic tricks; making sense of—or even just accessing—this knowledge is often challenging for both magicians and non-magicians alike.

One way to handle this is via a taxonomy. These are central to many scientific domains, aiding our understanding in fields such as chemistry, biology, and even mineralogy. If we intend to truly understand any aspect of magic—including misdirection—a taxonomy must be a crucial part of this endeavor (Rensink and Kuhn, under review).

Previous taxonomies of misdirection were developed from the perspective of magic performance (Leech, 1960; Ascanio, 1964; Randal, 1976; Bruno, 1978; Sharpe, 1988), or were based on rather informal psychological principles (Lamont and Wiseman, 1999). The central aim of our effort is to develop a more rigorous and less subjective system, one based as much as possible on known psychological mechanisms. Among other things, this approach can help draw more direct links between practical principles and current scientific understanding of the human mind.

## Previous taxonomies of misdirection

Magicians and scholars have written about misdirection for centuries; a full history of this is beyond the scope of the discussion here. Instead, we will simply review several of the more popular taxonomies which have been proposed; in particular, we review those based on relatively abstract principles, so as to highlight those principles to non-magicians. (Note that some of these taxonomies describe the same principles using different names.)

### Arturo Ascanio: Magical Atmosphere

In 1958 Arturo de Ascanio published a book which changed the way magic was understood. Ascanio was not the first to do so (e.g., Houdin, 1877; Fitzkee, 1945), but his was a particularly clear and systematic approach. Titled “Conception of the Magical Atmosphere,” one of its cornerstones is misdirection, included within a set of techniques about how to cover the secret of a magical effect. This set uses what Ascanio called the Principle of Coverage. Here, *coverage* refers to the “defense mechanisms” used by the magician to hide the method of any magical effect. In the words of Ascanio: “[its goal is to] ensure that the secrets are not shown, not known to exist, not even suspected” (Etcheverry, 2000d, p. 35).

**Figure 1:**
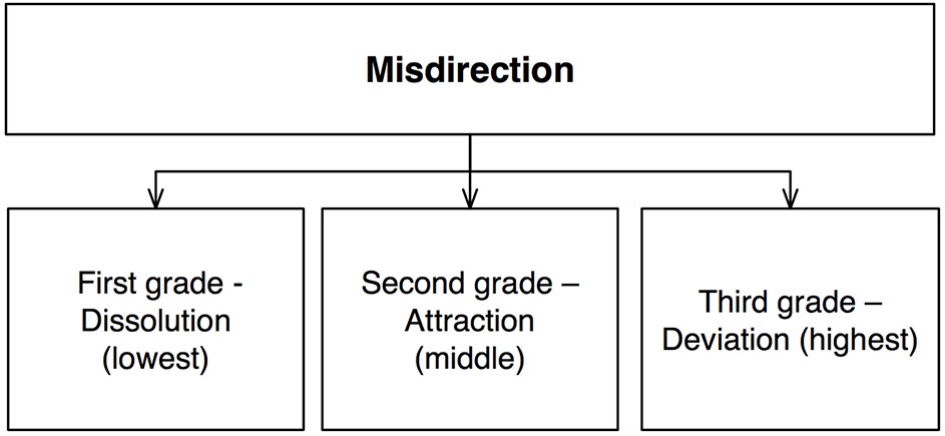
**Schematic description of Ascanio's (1964) taxonomy**.

Ascanio highlighted not only the importance of understanding the psychology of the spectator (misdirection, timing, etc.), but also that of the magician (naturalness, fluency of movements, handling, and so on) (Pareras, 2011). He defined misdirection as “the art of drawing the eye and the attention of the public to a safe and interesting point, while elsewhere a secret action, which is therefore invisible and unsuspected, is carried out” (Etcheverry, 2000b, p. 47). However, he later noted (Ascanio, 1964) that this definition was in fact “poor,” since misdirection could have three different grades, or levels of intensity (Figure 1):

#### First grade—dissolution (lowest)

This is achieved by giving the spectator two distinct points of interest: the secret, along with an innocuous other event. The spectator's attention is thereby divided and their experience of the secret “dissolved,” since it is impossible to completely attend to two different points at the same time.

#### Second grade—attraction (medium)

Here, the innocuous point of interest is more attractive to the spectator than the secret one. It therefore grabs their attention away from the method/secret, effectively removing any real experience of its structure.

#### Third grade—deviation (highest)

This is achieved by a total deviation of the gaze and attention of the spectator to the innocuous point of interest. This results in a complete absence of visual experience of the remainder of the scene, including the secret.

When these techniques succeed, attention is focused on the innocuous point of interest, known as the “illuminated” area, with the secret remaining in the “shadowy” area (the lower attention area). This is what Ascanio called the Tube Effect (Etcheverry, 2000c, p. 78), comparable to the spotlight metaphor of attention (Posner, 1980). These areas (illuminated and shadowy) could be physical or mental, as there may be a mental distraction (a question, or something to make the spectator think about, and that would be a “illuminated area”) while the secret action is performed in the shadows^2^.

Later authors in the world of magic built on Ascanio's work. As an example, Randal (1976) discussed five types of misdirection. The first is *Misdirection of Attitude*, whereby the magician marks the points of interest with his gaze and attitude. Second is *Misdirection by Transfer* (comparable to the manipulation in the third grade of Ascanio's theory), in which the magician directs the attention of the spectator, using gestures and glances, toward a point far away from the place where the magic secret is happening. Third is *Misdirection by Repetition*, which accustoms the spectator to a specific gesture (by repetition) in order to relax their attention when that gesture performs the secret movement (Etcheverry, 2000a). Finally, he differentiates between *Verbal Misdirection*, which emphasizes the speech of the magician (to distract the attention), and *Non-Verbal Misdirection*, including the gestures, personality, and attitude of the magician.

### Joe Bruno: Anatomy of Misdirection

**Figure 2:**
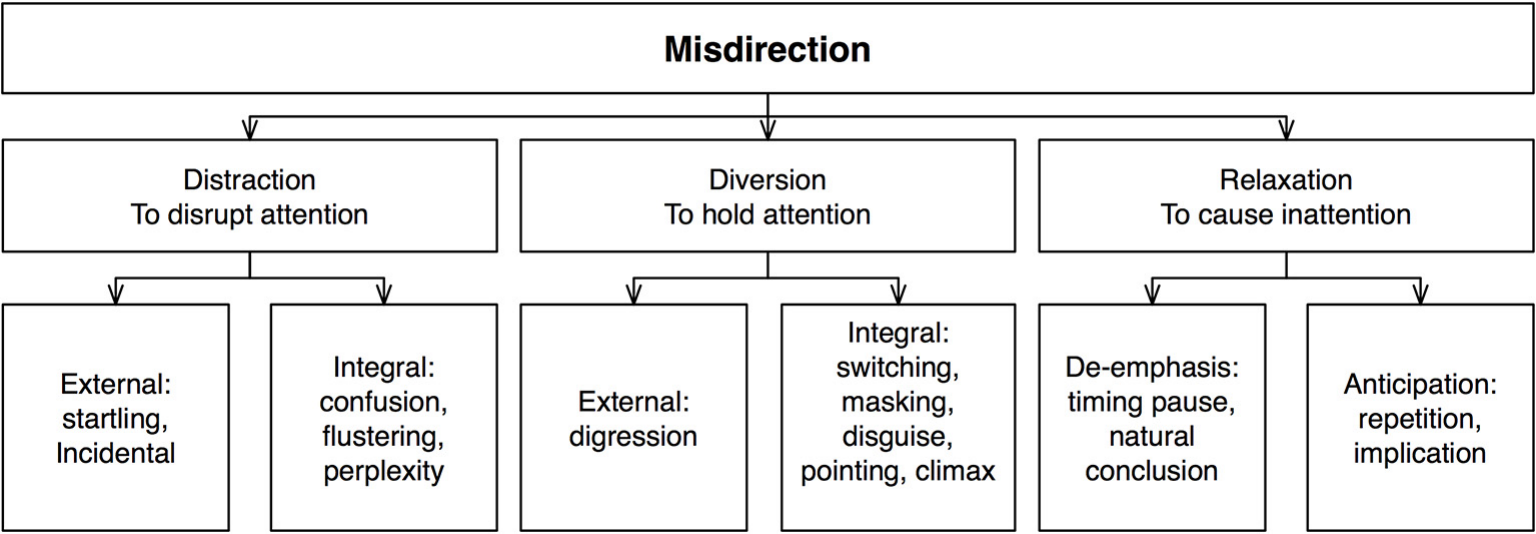
**Schematic description of Bruno's (1978) taxonomy**.

In 1978 Joe Bruno wrote a book titled “Anatomy of Misdirection,” aimed at teaching magicians the ways in which attention can be manipulated (Bruno, 1978). Possibly inspired by Buckley (1948), his approach focuses on three distinct kinds of technique: distraction, diversion, and relaxation (Figure 2).

#### Distraction

*Distraction* refers to situations in which several things occur at the same time. The premise here is similar to that of Ascanio: people can only process a limited amount of information at any moment, so if their attention is distracted by one event they will not notice anything in the unattended location(s). According to Bruno, one type of distraction is *external* to the proceedings, generally taking the form of an unexpected event such as an interruption. This can range between crude and subtle. An example of a crude external distraction would be a loud bang. This is extremely effective but can easily disrupt the performance, and so diminish the effect. Consequently, magicians usually opt instead for subtler forms, such as a well-timed cough.

In contrast, *integral distractions* are core parts of the performance. According to Bruno there exist three types: confusion, flustering, and perplexity. *Confusion* can potentially occur during various parts of a performance; for instance, when the magician asks a spectator to join him on stage. Such moments offer valuable opportunities to execute a method, such as switching a deck of cards. *Flustering* can be achieved by asking the spectator a difficult or potentially embarrassing question; not only does this distract the person, but it ensures that the rest of the audience focuses their attention on the spectator, and thus, away from the magician. Finally, *perplexity* occurs in a situation that is either complicated or puzzling to the spectator. This is rather challenging to create, as there is a fine line between confusion and boredom, and the latter should be avoided at all cost.

#### Diversion

If people become aware of being distracted, it can take away from the effect, which is why distraction tends to be considered a suboptimal technique. Instead, magicians generally prefer *diversion*, which differs from distraction in that only one thing appears to be going on. Like distraction, diversion can be either external or integral to the performance. *External* diversions are digressions where attention is oriented away from the method via an apparently unconnected event. For example, the magician may use an amusing interlude that captures the audience's attention and thus allows the magician to execute his secret method unnoticed. Meanwhile, *integral* diversions are built into magic tricks themselves.

Bruno identified five types of diversion. *Switching* refers to the side-tracking of attention from one area of interest to the other—e.g., each time the magician produces a new prop, attention switches to this new object. Next is *masking*, whereby one action screens another. For example, the magician may change his body orientation so that the view of his hand going to his pocket is obstructed or at least becomes less salient. The third principle is *disguise*, where an action appears to be performed for one purpose when in reality it is done for another. For instance, the magician might reach into his pocket to pull out a scarf when in fact the action is used to deposit a secret prop. Related to this is the idea that large motions will disguise small ones. Fourth is *pointing*, where the magician pauses for a dramatic emphasis. A method must be executed either before or after these pauses, to avoid detection. Finally, one of the strongest diversions of attention can be created by using the *climax* of an effect. This offers an ideal moment at which the method for the next effect can be executed. For example, in the Cups and Balls routine, small climaxes such as when the balls appear or disappear offer ideal diversions of attention that allow the magician to prepare for the next effect.

#### Relaxation

Bruno's third general principle is *relaxation*; this relates to the temporal fluctuations in attention created though off-beat moments in a routine. For example, attentional *de-emphasis* can occur once a magic trick has been concluded: if the magician picks up a bowl in preparation for his next trick, say, the audience won't suspect the execution of the method at that time. Meanwhile, *anticipation* can get spectators to relax their attention because they think they know what is going to happen. Relaxation can also be created through *repetition*, whereby the magician repeats an action several times, so that the spectator will pay less attention to the subsequent action (Bruno, 1978; Kaufman, 1989).

Bruno's taxonomy provides valuable insights that can help magicians think about attentional misdirection. However, it has two serious limitations. First, it relies on a rather narrow definition of misdirection in terms of attention, and so does not discuss ways of manipulating what people remember, or how they interpret an event. In addition, Bruno's approach was written for magic practitioners, and so does not directly link his principles with known mechanisms of perception and cognition.

### Sharpe: Conjuror's Psychological Secrets

**Figure 3:**
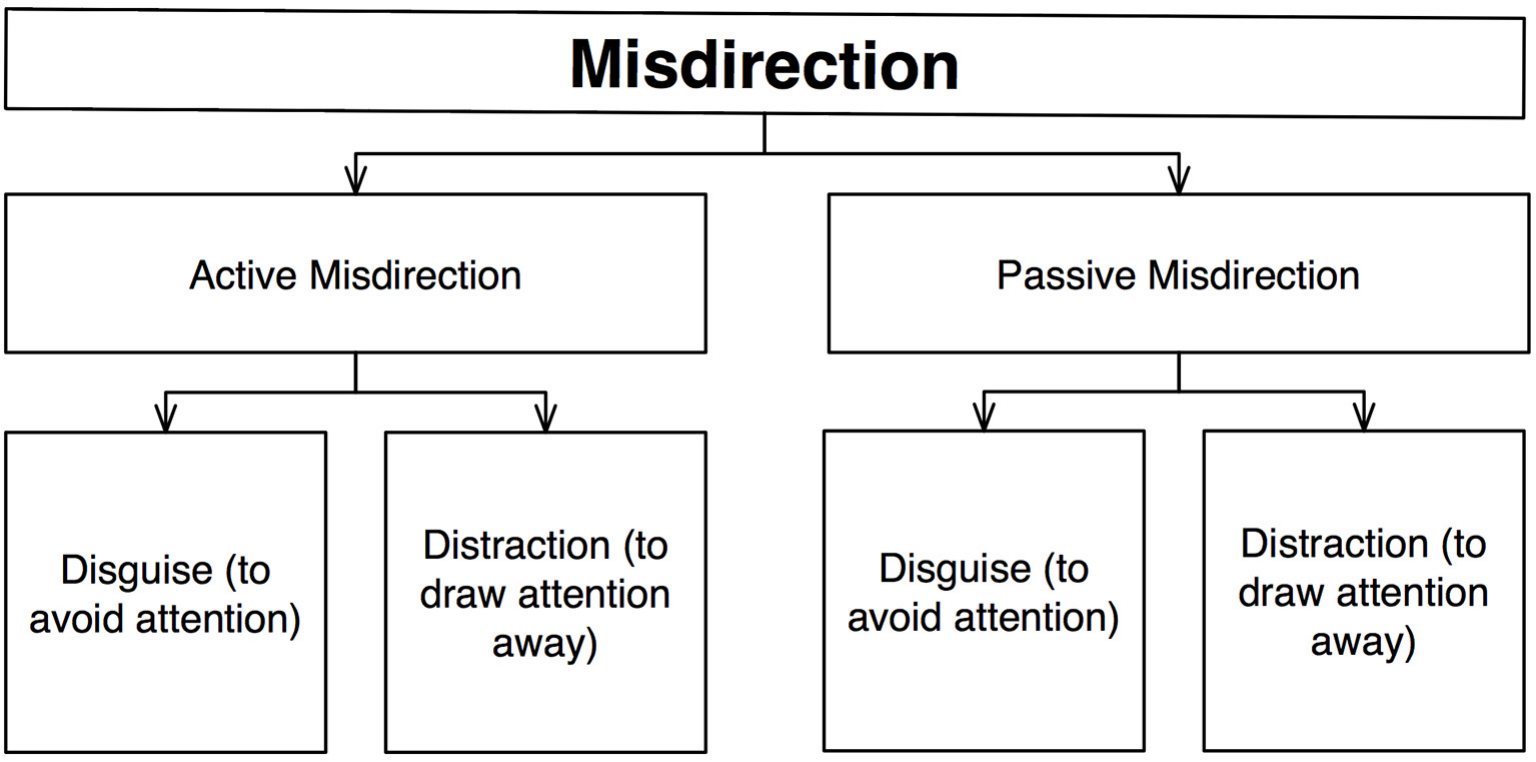
**Schematic diagram of Sharpe's (1988) taxonomy**.

Sharpe (1988) published a book entitled “Conjuror's Psychological Secrets” that attempted to systematize much of the psychological basis of conjuring (Figure 3). Its main focus is on misdirection, defined as the “intentional deflection of attention for the purpose of disguise” (p. 47), a definition that again heavily relies on attention.

Sharpe divides misdirection into two kinds: *active*, which covers methods that depend on “*some kind of change in movement or sound*” (p. 47), and *passive*, which covers methods where “*misdirection works unobtrusively on the spectator's mind, owing to an understanding of how the mind reacts to given static stimuli*” (p. 47). Within each of these, misdirection can either *disguise* something “by altering its appearance in some way, so that casual attention fails to focus on it owing to lack of interest” (p. 47), or *distract* the spectator by focusing their attention “elsewhere by introduction a more powerful stimulus to act as a decoy” (p. 47).

Sharpe classified a wide range of misdirection methods in terms of these four categories. For example, when magicians familiarize the spectator with actions or objects, people relax their attention and so become less aware of otherwise suspicious behavior. This principle is categorized as active misdirection for disguise since it prevents people from attending (disguise) to the novel action (active). Active misdirection for distraction often includes audience participation, e.g., asking a person to join the magician on stage (active) draws attention away from the magician and toward the volunteer (distraction). Other forms include the use of patter (i.e., spoken presentation), or different kinds of movement. Meanwhile, passive misdirection for disguise includes principles such as camouflage that makes an object unnoticeable by obliteration, or immobility that cause disregard though lack of movement. And passive misdirection to distract includes the principle of novelty that can be used to stimulate curiosity by presenting something unusual or unfamiliar.

Sharp's inventory is a useful starting point for a more psychologically-based categorization of distraction techniques and principles. However, his analysis is somewhat disjointed (e.g., he simply lists numerous concepts), and many key concepts are loosely defined. For example, whilst misdirection is defined in terms of attentional strategies, several non-attentional principles are also included (e.g., those concerned with memory, reasoning). More importantly, perhaps, few links are made to formal psychological mechanisms. For example, misdirection is defined solely in terms of attentional processes, and although non-perceptual processes are described (e.g., memory), little attempt is made to distinguish them from perceptual ones. And whilst the distinction between distraction and disguise seems intuitive, the same cannot be argued for active vs. passive misdirection^3^.

### Lamont and Wiseman: Magic in Theory

**Figure 4:**
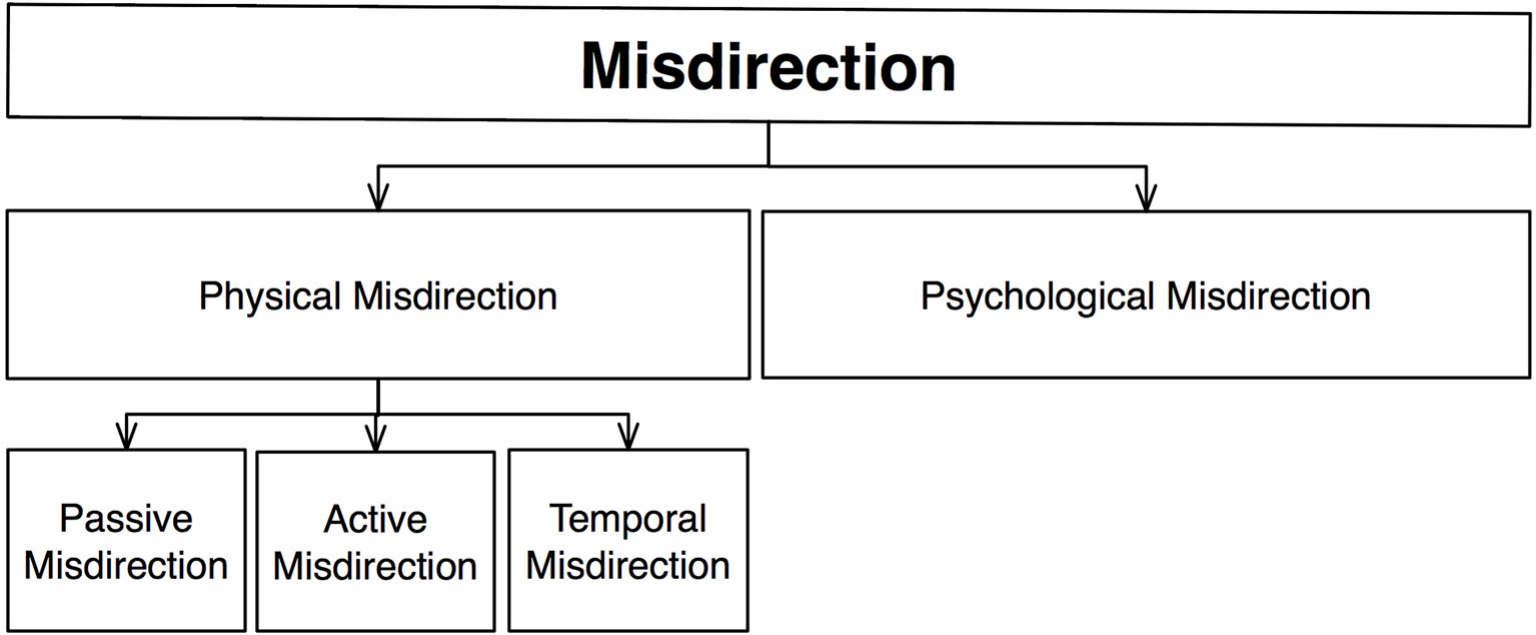
**Schematic diagram of Lamont and Wiseman's (1999) taxonomy**.

A more recent taxonomy is that of Lamont and Wiseman (1999), who discuss various theoretical and psychological elements of magic in their book “Magic in Theory” (Figure 4). Although both authors are academics, they avoid making direct links with academic psychology; their framework is intended to focus on how magic is understood by magicians rather than scientists.

Lamont and Wiseman define misdirection as “that which directs the audience toward the effect and away from the method” (p. 31), extending its scope beyond the simple manipulation of attentional processes. They present a simple taxonomy of misdirection that explicitly distinguishes between attentional and non-attentional processes, which are affected by what they define as *Physical* and *Psychological* misdirection, respectively.

*Physical* misdirection deals with manipulating people's focus of attention: “what the spectator perceives is determined by where and when the spectator is looking, i.e., where and when the spectator's attention is focused” (p. 37). It is based on the idea—similar to that proposed by others—that magicians create areas of high interest, thereby preventing the spectator from noticing things elsewhere. Three kinds of misdirection are distinguished, involving passive, active, and temporal diversions of attention. The first of these, *passive* misdirection, uses any property that attracts attention in its own right—e.g., novelty, or sudden changes in pace or facial expressions. Contrast is another important example, whereby objects that differ from their background will attract attention (e.g., bright colors that stand out).

Meanwhile, *active* misdirection relies on social interactions created by the magician's actions. For instance, the magician may use his eyes to direct attention toward looked-at areas, or use his voice (through patter) to create interest in certain objects; in some cases the magician might simply instruct a spectator to look somewhere. Another form of active misdirection involves body language, which can convey non-verbal information to direct attention. The magician may also use an external source of diversion, such as the actions of an assistant or a member of the audience.

Lamont and Wiseman note that just as people tend to vary their level of attention throughout *space*, they also tend to vary their level of attention throughout *time*. The magician may therefore create *moments* (as well as locations) of primary and secondary interest—for example, people are less likely to pay attention if they believe that the trick has not yet begun, or is already over. Temporal fluctuations may also be exploited. For example, repetition can lead to tedium, which reduces the spectator's level of interest, and therefore, attention. Alternatively, the magician may create an off-beat moment through a momentary relaxation, such as after a joke (Tamariz, 2007) or a magical effect. These off-beat moments are thought to reduce attention, and thus allow the magician to execute the method without being noticed. Magicians may also use their body to create moments of tension and relaxation (Ganson, 1980; Kurtz, 1989).

In contrast, *psychological misdirection* involves manipulating people's suspicions^4^. Seeing a method clearly provides strong evidence of its use, but there are many situations in which a method may not have been seen, but is still suspected. Magicians often talk about the need for actions to appear *natural*, as anything unnatural will generally arise suspicion. For example, in the French Drop the magician pretends to pass a coin from one hand to the other whilst retaining the coin in the original hand (Supplementary Video 1). If this false transfer appears unnatural, it will arouse suspicion and thus attract unwanted attention, resulting in its detection.

Lamont and Wiseman also discuss ways in which magicians divert suspicion by misrepresenting the method. One of the most powerful tools for this involves deliberately raising suspicion about a *false solution* which will distract from the real solution. This can be applied to differing degrees (Tamariz, 1988). An extreme form is the “sucker trick,” in which the magician presents an obvious yet false solution that is later revealed to be wrong. For example, in the Egg Bag trick, an egg appears and disappears inside a cloth bag. In the standard routine the magician pretends to sneak the egg under his arm, after which he shows the bag to be empty. The real method involves a secret compartment inside the bag that allows to magician to conceal the egg; when the bag is shown empty, it attracts little attention, since the audience thinks it knows where the egg is. More subtle ways of leading the audience down the garden path are also possible (e.g., Tamariz, 1988).

Lamont and Wiseman's taxonomy of misdirection is a great improvement on earlier efforts because it makes several important links between magic theory and human cognition. However, it lacks scientific rigor, and some of the categories still seem rather arbitrary. For instance, looking and seeing (or at least, attending) are treated as equivalent. However, this is not the case: research has shown that you can look at things without seeing them (Mack and Rock, 1998); indeed, eye movements are only one of several forms of attention, which are not always co-ordinated with each other (Rensink, 2013). Several other category divisions are also rather problematic. For example, the terms “active” and “passive” are misleading, and do not necessarily refer to mutually exclusive processes: many passive misdirection principles, such as movements, require actions, and it is difficult to see how this could be considered anything other than active. More generally, many of the terms and categories are rather vague, and not always based on recent scientific models of cognition. A taxonomy that is to help create connections between magic and science should be based as much as possible on our current understanding of perception and cognition.

## A psychologically-based taxonomy

The primary purpose of any taxonomy of magic is to organize the methods and effects used in known magic tricks. An important secondary purpose is to do so in a way that enables clear connections to be drawn between the tricks and the psychological principles they draw upon. To show how such a taxonomy might look, we focus here on the area of misdirection.

As a first step, we will describe magic tricks in somewhat abstract terms, focusing on the general factors that govern their effectiveness, rather than the particular details of a performance. (Ideally, however, both abstract and concrete taxonomies would be possible—cf. Rensink and Kuhn, under review). And rather than a taxonomy based directly on the particular methods used or effects created, we propose one that arranges these (in their abstract form) according to two fundamental taxonomic principles. First is the *principle of maximal mechanism*: the taxonomy should be based as much as possible on known psychological mechanisms and principles. Second is the *principle of effect priority*: the highest levels of the taxonomy are those involving the mechanisms being affected (i.e., those underlying the effect); the mechanisms controlling these (i.e., those underlying the method) are secondary, relevant only after the first set has been exhausted. Other considerations (e.g., aspects of the performance) can still be included, although these would be relevant only for those categories at the lowest levels. An important advantage of this approach is that we can borrow well-established terms and concepts from the behavioral sciences, and so avoid many of the complications arising from vague or arbitrary categories. Moreover, it makes the connections with known psychological mechanisms quite clear, facilitating interaction between magicians and researchers. Finally, it also minimizes the effect of subjective elements in the structure of the taxonomy, opening up the possibility of a system that might be accepted more generally^5^.

To see how such a taxonomy can be developed, begin by noting that human cognition generally involves several different kinds of information processing: when confronted with a magic trick the observer first *perceives* the relevant sensory information, *stores* key aspects of it in memory, and then perhaps uses this information to *reason* out how the trick was done. To prevent a spectator from discovering the method, a magician could manipulate any of these processes (Kuhn and Martinez, 2012).

**Figure 5:**
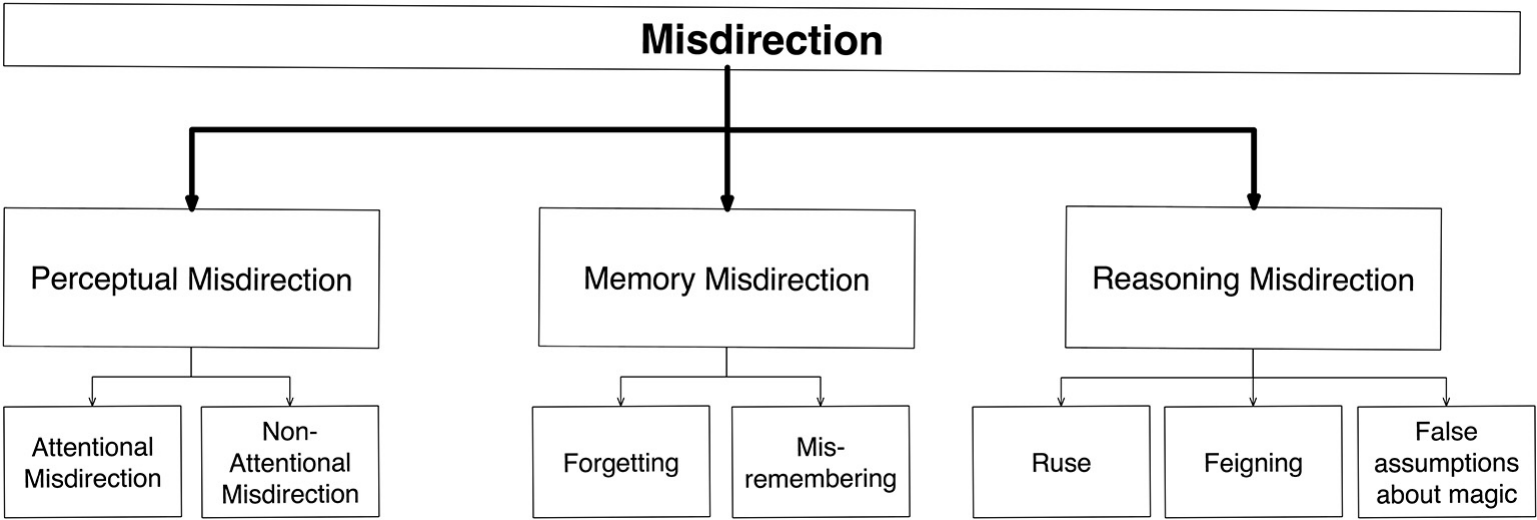
**Schematic diagram of the psychologically-based taxonomy, showing its highest levels**. Here, divisions are based on the mechanisms underlying the effects involved.

Our taxonomy therefore has three broad categories, corresponding to the three broad kinds of mechanisms affected (Figure 5). The first encompasses those procedures that manipulate *perceptual* mechanisms, preventing you from noticing particular events. Even if an event is perceived accurately, however, there is no guarantee you the spectator will accurately remember it later on—our memories are very selective, and based on reconstructions of fragments rather than complete representations of objects or events (Fernyhough, 2012). Our second category therefore involves *memory*. But even an accurate memory of a magic trick does not guarantee the spectator will discover the method if he/she cannot bring to bear correct *reasoning*. Thus, the third category of misdirection relates to manipulating the way that people reason about an event^6^.

Although these kinds of process operate separately to a large extent, they are nevertheless interdependent. (This reflects the interdependent operation of perceptual and cognitive mechanisms generally). For example, our perception of an event influences what we remember, and our memories in turn guide our reasoning and attention. Moreover, certain misdirection principles can potentially influence cognitive functions at multiple levels. In such a situation, however, their components could be separated out, and the principles treated as “compounds” composed of more basic units.

We next discuss these three categories in more detail:

### Perceptual misdirection

This refers to misdirection that manipulates the perception of an event. This category is somewhat similar to Lamont and Wiseman's physical misdirection, except that their category includes only attentional processes^7^, and so ignores non-attentional factors such as occlusion. Most importantly, however, unlike their physical misdirection, the categories here are centered around a well-founded and well-articulated set of perceptual and cognitive mechanisms.

A large number of misdirection techniques fall under this category. The most basic division is that between *attentional* and *non-attentional* mechanisms (Figure 4). This distinction has important theoretical and practical implications. For example, most attentional effects can be modulated by direct top-down control, which is not necessarily the case for non-attentional ones. Among other things, this highlights that the misdirection of non-attentional perceptual mechanisms is more resilient to the spectator's own intentions.

#### Attentional misdirection

**Figure 6:**
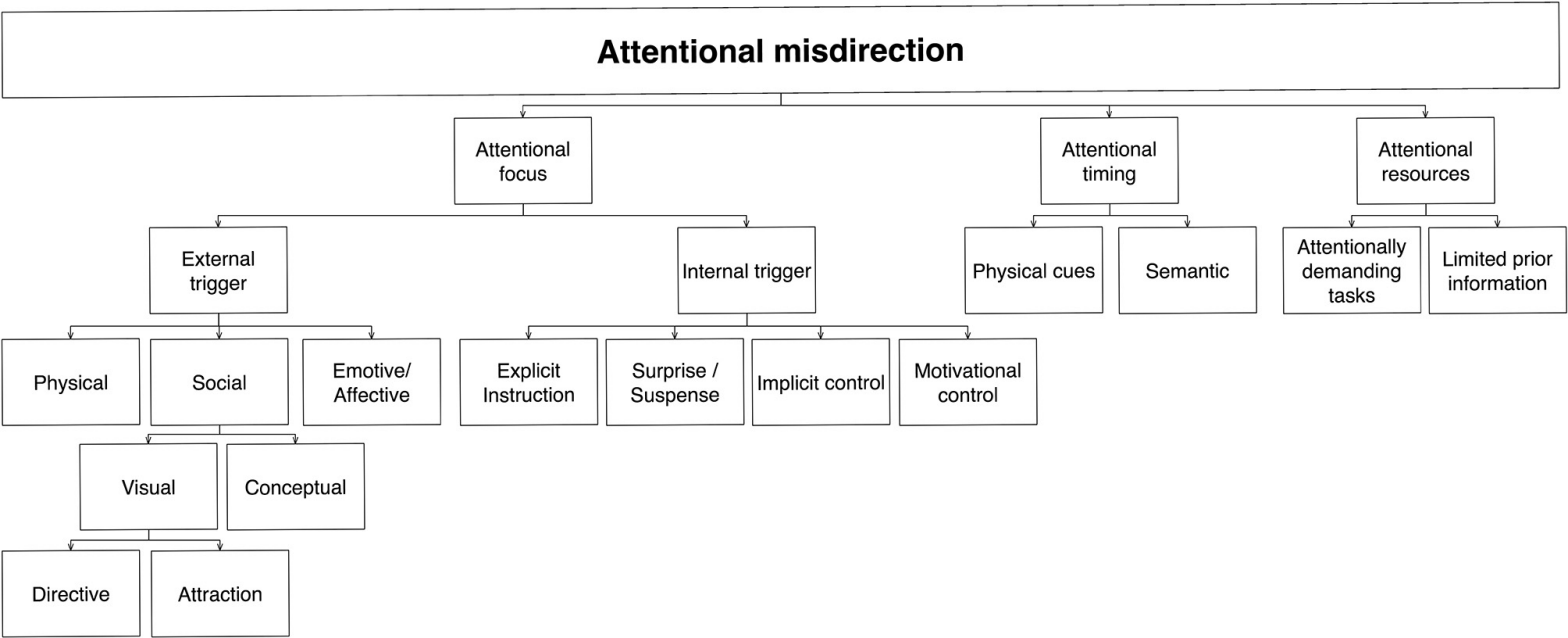
**Schematic diagram of attentional misdirection**. Here, the initial level is based on the mechanisms affected (focus, timing, capacity). Later divisions are based on the mechanisms that underlie the methods involved.

Given the central role of attentional processes in creating our conscious experience (e.g., Kuhn et al., 2008a; Rensink, 2010), it may not be a surprise that their manipulation is the goal of the largest group of perceptually-based misdirection techniques (Figure 6). Attention is a notoriously difficult phenomenon to define; among other things, it is currently unclear how many attentional process there are, or exactly what each of them does (see e.g., Rensink, 2013). But whatever characterization is used, there appear to be three distinct aspects of attention that can be manipulated, each involving a distinct set of mechanisms:

1. Attentional *focus*, which describe what you are attending to.
2. Attentional *timing*, which describes when you pay attention.
3. Attentional *resources*, which describes how much attention is given.

Note that subdivisions below this level are method-centered—i.e., focused on “hijacking” the mechanisms that control the processes underlying each of these three aspects (cf. Rensink and Kuhn, under review). As for other parts of this taxonomy, we expect that future research may well uncover other aspects of attentional control, which would correspondingly give rise to new subcategories in the taxonomy.

***Control of attentional focus***. This refers to *what* is attended—e.g., a particular object, or a particular region of space. Many concepts of misdirection refer to manipulating this aspect either explicitly (Bruno, 1978; Lamont and Wiseman, 1999), or implicitly through creating zones of high and low interest (Sharpe, 1988). Techniques where the magician orchestrates spatial attention are all grouped in this category. Such misdirection can be divided into two main subgroups: those triggered externally (i.e., reflexive, or exogenous control) and those triggered internally (i.e., contextual, or endogenous control).

*External (reflexive) triggers*. External triggers cause attention to be controlled as a reflexive result of events in the environment—for example, a bright flash. Such control can be further subdivided into procedures involving physical, social, and emotive processes.

- *Physical*. These techniques send attention toward objects or events based on their inherent physical properties. For example, we generally attend to objects that are *visually salient*, such as a bright light (Kuhn and Tatler, 2005) or a blue card amongst a set of red cards. The capture of attention by the appearance of a new object (Yantis and Jonides, 1984) also forms the basis of many misdirection techniques. Such techniques need not be limited to the visual domain: an *auditory event* such as a loud sound, or a somatosensory event such as a light touch can also control attention.
- *Social*. Another form of attentional control involves *social* interactions between the magician and his audience; these are based on overlearned responses that are effectively automatic. Both visual and conceptual forms exist. *Visual* social cues can send attention toward or away from selected locations or objects via social *directives* (Kuhn et al., 2009). For example, the magician may change his *facial expression*, or establish *eye contact* to draw attention toward himself (Tamariz, 2007); if attention needs to be directed away, he might use *head, eye gaze, pointing or body postures* (Ganson, 1980; Kurtz, 1989). Another powerful visual social cue that attracts attention is to bring another person—especially a child—on stage (Bruno, 1978). All of these cues are visual since they result directly from perceiving a visual signal.
  Social directives can also act on a conceptual level, where some degree of interpretation is involved. For example, asking someone a question, or requesting the persons' name, are powerful tools to draw attention to the magician (Kurtz, 1989; Tamariz, 2007). Actions that fluster a participant (such as asking embarrassing questions) can—if used in small doses—also draw attention toward the flustered person (Bruno, 1978). A similar effect is achieved by using *confusion* to draw attention away from the magician (Bruno, 1978).
- *Emotive (or Affective)*. These are stimuli which are likely to capture your attention via the emotions they induce (Vuilleumier and Schwartz, 2001). This dimension is frequently exploited by magicians. For example, the production of a cute rabbit is highly likely to capture the audience's attention.

*Internal (contextual) triggers*. Although our attention can be captured by external events, we also have some degree of conscious control over where we attend—such as when you decide to attend to a particular location in a scene (Posner, 1980). Many misdirection techniques influence these processes by manipulating internal goals or intentions, typically via narrative.

- *Explicit instruction*. The most explicit form of this involves the magician asking you to attend to something, e.g., a set of cards being shuffled. Such misdirection is very effective, but is likely to be noticed, and so raise suspicion. Rather than explicitly instructing you to attend to a particular location, then, a better approach is to ask you to do some task, one that requires your attention—for instance, shuffling a deck of cards or writing something down on a piece of paper. These types of instructions commit your attention to the task and prevent you from attending elsewhere.
- *Surprise/suspense*. Another effective manipulation is the use of *surprise*. By definition, surprise is determined by your expectations about the immediate future; magicians can manipulate context to create many surprising events that are very effective at capturing attention. For example, Blackstone had a technician chase a duck that escaped from a box. Whilst the audience focused their attention on the technician, another person removed the remaining ducks from the box without being noticed (Leech, 1960).
  Related to this is the creation of *suspense*. This ensures that you attend to the object or event in question, thereby preventing any search for alternative explanations. For example, imagine that a coin is held in one hand and the magician explains that he will vanish a coin the third time it is struck by the magic wand. The expectation that the coin will vanish creates considerable interest in the coin and so focuses people's attention on it. Then, instead of vanishing the coin, the magician uses the misdirection to vanish the magic wand (Supplementary Video 2).
- *Implicit control*. One of the more powerful principles in misdirection involves the use of *implicit (i.e., unnoticed) suggestions* to essentially hijack the orienting of attention (see e.g., Rensink and Kuhn, under review). For example, magicians often use *patter* to talk about certain objects or events, resulting in your attention being sent there without you being aware of it. Implicit suggestions can increase or decrease the level of attention given to something. For example, magicians may reduce your level of attention by making an object or event seem mundane. For example, in the coin vanish described above (Supplementary Video 2), magicians typically carry out the method on the third strike, when events seem less novel (Kaufman, 1989). Another principle that falls within this category is the idea that people are less likely to attend to *justified* rather than unjustified actions (Lamont and Wiseman, 1999). Similarly, sucker tricks and the theory of false solutions can influence attentional processes in that we simply pay less attention toward alternative solutions.
  Much of implicit control relies on naturalness. Magicians repeatedly state the importance of actions and props that seem natural in order to avoid suspicion, and therefore, attention (Ganson, 1956; Lamont and Wiseman, 1999). Whether something is natural or not depends on the event itself as well as the context in which it occurs. For example, palming a card always results in a rather unnatural hand posture, but the posture will seem much more natural if the hand is holding a glass at the same time. Lamont and Wiseman classify techniques relating to naturalness as part of psychological misdirection. However, as these principles work on attentional mechanisms, we consider them part of perceptual misdirection.
- *Motivational control*. Another powerful principle is to control the *motivation* of the spectator to search for a method. For example, a poorly motivated person is less likely to seek out the method, and so more likely to attend to things the magician does not want them to see (Lamont and Wiseman, 1999). Other principles relate to the magician's persona or expertise: if the magician is more likeable, for example, you are less likely to want to trip him up by attending to the wrong location. One of the most skilled card magicians, Lennart Green, often pretends to be incapable of handling playing cards, reducing the motivation of the naïve spectator to search for expert sleight of hand.

***Control of attentional timing***. Just as we can focus our attention on particular objects or locations in space, so can we focus it on particular moments in time. Magicians have accordingly developed several types of techniques that manipulate how much attention is paid at a particular time within a magic trick. Such control is similar to the temporal misdirection of Lamont and Wiseman (Section Lamont and Wiseman: Magic in theory), except that our taxonomy prioritizes the mechanisms, rather than the methods by which the misdirection is achieved. People's level of attention can either be manipulated through *physical cues*, or by exploiting fluctuations in attention that naturally occur during the performance, and require a *semantic* understanding of the performance.

- *Physical cues*. Magicians have techniques to control the level of attention, many of which rely on physical cues. Slydini, a master in misdirection, developed body postures that led to tensions and relaxations in attention (Ganson, 1980). For example, forward postures will result in tension and thus heighten people's level of attention, whilst leaning back is an apparent relaxation and reduces the level of attention.
- *Semantic*. Other techniques rely on an understanding of the performance; thus, they are often categorized as *semantic* techniques. People are less likely to pay attention to things just after they have experienced the climax of a routine. For example, in the Cups and Balls routine, people are less likely to notice the magician's hand going into his pocket just after he has made a ball appear (Ganson, 1956). Humor can also act as a powerful misdirection technique whereby people are less likely to spot the method if it occurs immediately after the *joke*. These off-beat moments can also be created by the magician making an aside to the audience, as in the moment the lighter is ditched before being vanished (Supplementary Video 3).
  One of the most powerful misdirection techniques involves carrying out the procedure before the effect has started, largely because most people do not expect the method to take place outside the effect. For example, the magician could vanish a lighter by apparently eating it, and the method is simply that the lighter is already out of his hands before he “eats” it (Supplementary Video 3) (this is similar to the pen being out of the magician's hands before the “vanish” motion in Demacheva et al., 2012). Meanwhile, other magic tricks require methods that are carried out after the effect. Again, such procedures rely on the fact the people do not expect the method to be conducted outside the effect, and so pay less attention to them.

***Control of attentional resources***. The perception of information depends not only on available information, but also on the *attentional resources* available. People engaged in an attentionally-demanding task often fail to notice extremely obvious events that occur directly in front of them (Mack and Rock, 1998; Chabris and Simons, 2009). Several types of misdirection are therefore based on manipulating the attentional resources available. The most explicit involves explicitly giving someone an attentionally-demanding task. For example, the magician might ask a person to count the number of face cards among those being dealt onto the table. Since their attentional resources are occupied by this, they will fail to notice things going on elsewhere (Smith et al., 2013). A related form of this—which also plays a central role in Bruno's taxonomy (Section Joe Bruno: Anatomy of misdirection)—is the creation of *confusion*. If lots of different things are going on at the same time that require a lot of attention, the spectator will be prevented from encoding much of the detail. (Of course this only works as long as they can still follow the trick.)

One of the key rules in magic is that you should never repeat the same effect with the same method. Indeed, empirical work confirms that people are less effectively misdirected if the same trick is repeated (Kuhn and Tatler, 2005). This is likely because perceiving something for the first time requires more attentional resources than when you experience it a second time, a phenomenon known as *perceptual fluency* (Whittlesea and Leboe, 2000). For similar reasons magicians usually don't tell the audience what they are about to do; the level of suspense requires more attentional resources and thus prevents people from noticing the method (Kuhn et al., 2008b).

#### Non-attentional misdirection

**Figure 7:**
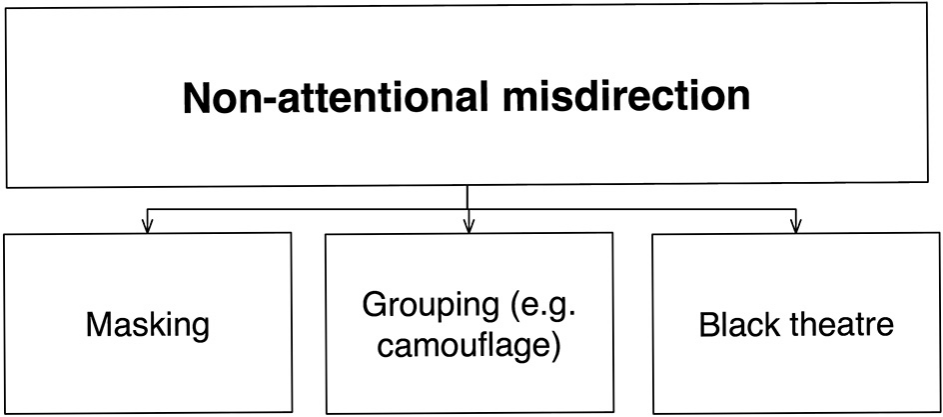
**Schematic diagram of non-attentional misdirection**. Here, the mechanism affected is non-attentional perception (largely based on perceptual organization, although further distinctions might be made 1 day). Categories are based upon the various ways to control this.

In addition to attention, our perception of a stimulus is influenced by various other factors, such its visibility and the context in which it is presented. Non-attentional misdirection techniques control the processes involved with these factors in one form or other (Figure 7).

- *Masking*. In *masking*, people are prevented from perceiving an event by the presence of a physical occluder or competing event—for example, the magician may secretly put his hand into his jacket pocket whilst turning to one side, which then interrupts the spectator's line of sight (as used to vanish the coin in Supplementary Video 2). Such masking is not limited to the visual domain—magicians often mask an unwanted sound by playing loud music or talking loudly. Likewise, pickpockets often use tactile masks (such as a strong pressure on the wrist) to prevent the victim from noticing how they steal the watch.
- *Grouping*. Another form of non-attentional misdirection involves the control of *grouping* mechanisms. Magicians often use *camouflage* to prevent people from seeing important parts of their apparatus. For example in a levitation, the magician must ensure that nobody sees the ropes that suspend the lady; much of this relies on camouflage to prevent the segregation of the object (i.e., the ropes) from the background. In essence, these techniques control grouping (typically acting prior to the operation of attention) so as to result in perceptual groups that do not correspond to structures that exist in reality. A related set of techniques uses optical illusions to achieve the same result (Sharpe, 1985; Barnhart, 2010).
- *Black light theater*. Although traditionally not thought of as misdirection, the ancient art of black light theater is also part of non-attentional misdirection. Brightly-colored objects appear and disappear in front of a black background by being obscured with black cloth. Here the visual properties of fluorescent colors cause a failure to distinguish the various dark background items, making them appear to be part of a single undifferentiated void.

### Memory misdirection

**Figure 8:**
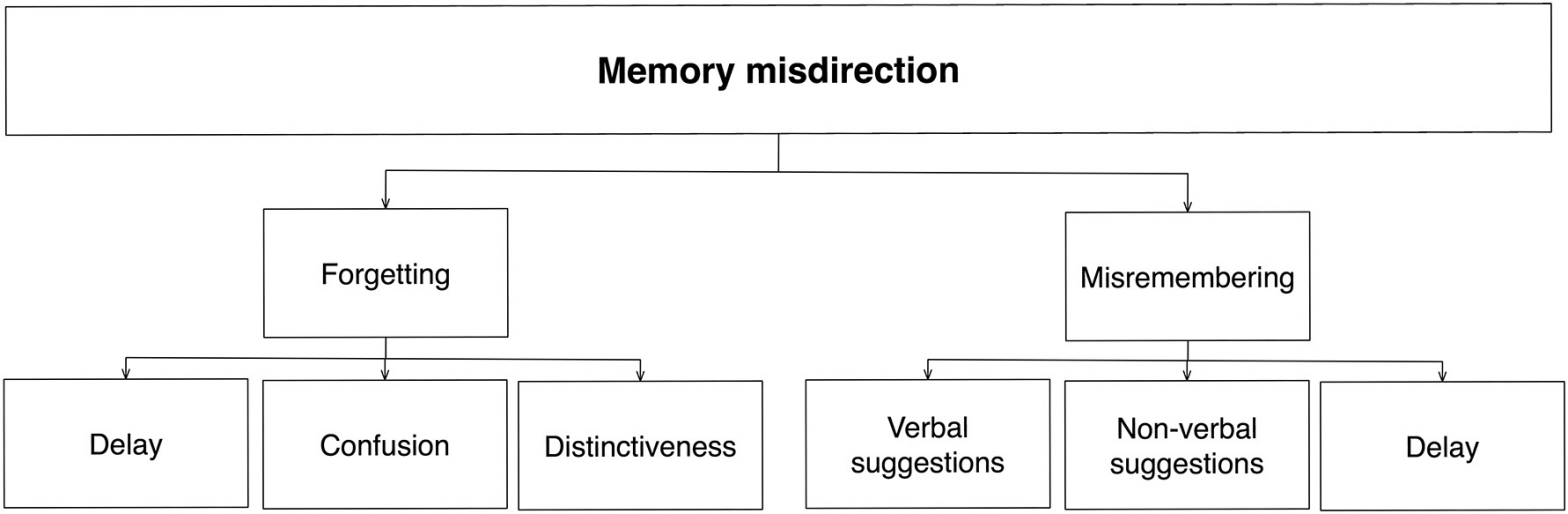
**Schematic diagram of memory misdirection**. The initial level is based on the mechanisms affected (maintenance, reconstruction). The divisions at lower levels are based on how these processes are controlled.

Our memories of an event depend not only on how well it has been perceived, but also on how well it has been retrieved. Memory processes are inherently reconstructive—you can easily misremember events that did not occur in real life (Fernyhough, 2012). *Memory misdirection* techniques can therefore affect the memory of an event by manipulating either the processes involved in its maintenance or in its reconstruction. Two distinct sets of techniques therefore exist: those based on *forgetting*, and those based on *misremembering* (Figure 8).

#### Forgetting

Many memory misdirection techniques try to ensure that relevant information about a magic method is simply forgotten. This can be done in several ways. For example, people remember more of an event immediately after it has occurred, as compared to some time later. The use of such *delays* is therefore an important kind of memory misdirection, and one of the reasons why magicians typically attempt to separate in time the method from the effect (Fraps, 2014; Leech, 1960). Leech calls this principle *time misdirection;* it is used in effects such as a prediction that relies on forcing a card (Supplementary Video 4) so that the spectator forgets which card he actually cut to. The extent of forgetting also depends on what the spectator is doing during the time delay; much is still unknown about what factors influence this.

Another important principle is the idea of *confusion*. Although akin to the similar concept used in other areas (attention), here it relates to the how the complexity of the environment affects memory: because our memory has a limited capacity, the more items there are, the less likely we will remember them all. There are several ways in which confusion can be created. For example, in card magic, magicians typically create magic routines that involve an entire deck of cards rather than a single card.

Confusion also helps prevent the audience from determining which details are relevant, further minimizing the chances that important parts of the method are remembered. A popular way of doing this is to provide the spectator with *false solutions*. These often take the form of pretending to carry out one effect whilst in fact doing something else (for example making a pen vanish after making it clear that they were attempting to vanish a coin, Supplementary Video 2). These techniques are often used to control attention, but they are also used to control memory: once we have a solution in mind, we are more likely to forget alternatives (Tamariz, 1988).

Related to this is *distinctiveness*. People are more likely to remember events that are distinctive; as such, magicians try to ensure that props or actions relating to the method lack distinctiveness, and thus will be quickly forgotten. This is typically achieved by either manipulating the props themselves or by manipulating the context and thus making them appear less distinctive and less likely to be remembered. For example, a mind-reading trick may require the spectator to write down a word; if the writing is done quickly on a bland scrap of paper that is used incidentally, the audience may forget that anything was ever written down.

#### Misremembering

Our memories are far less stable than we intuitively believe, with conscious recollection being based on a considerable degree on reconstruction rather than retrieval (Fernyhough, 2012). As such, the second category of memory misdirection involves the control of this reconstructive process to cause events to be misremembered. The most common form of this is people misremembering something as a *related* object or event, i.e., one similar to the original in key ways (Schacter, 2001). For example, we might see the magician perform an action that—at least to some extent—resembles a card shuffle; we later misremember it as a real shuffle. Consequently, misremembering is another fundamental principle in misdirection (Tamariz, 1988).

Another way to influence the contents of a reconstructed memory is by *suggestions*. These can be given before, during or after the event, and can be *verbal* or *non-verbal*. For example, verbal suggestions given at the time of a spoon bending resulted in people falsely remembering that the spoon was still bending whilst on the table (Wiseman and Greening, 2005). Similarly, visual suggestions that the magician threw a ball up in the air resulted in people falsely remembering that the ball was thrown (Kuhn and Land, 2006; Kuhn et al., 2010). Magicians likewise use post-event suggestions. A common technique involves the insertion of false claims when recapitulating the effect. For example the magician may suggest that the spectator, rather than the magician, shuffled the cards, in the hope that he/she will misremember a crucial detail, namely who it was that shuffled the cards (Giobbi, 1994); or suggest that the spectator cut to a particular card when in fact they cut to a different one (Supplementary Video 4).

A final way to increase misremembering is to increase the time lag between encoding and retrieval. As before, then, increasing the delay between method and effect are powerful ways of making it more likely that crucial aspects of the magic trick will be misremembered.

### Reasoning misdirection

**Figure 9:**
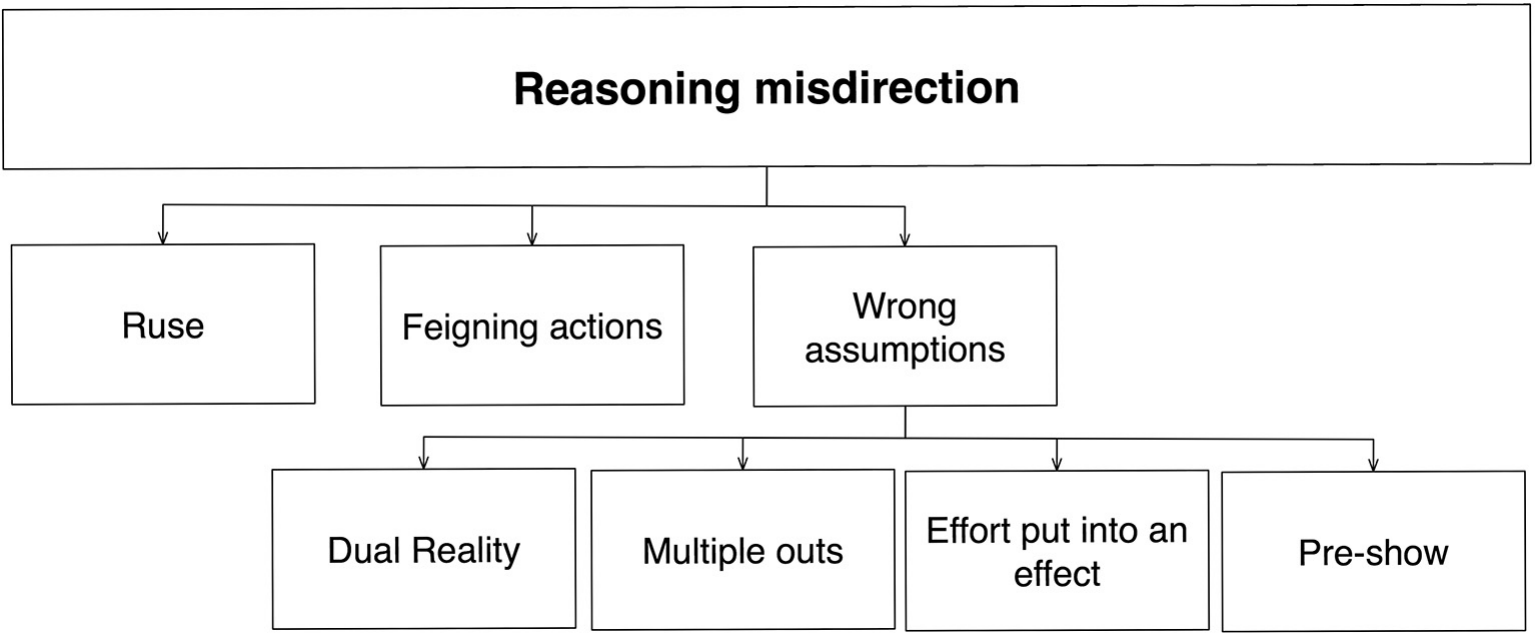
**Schematic diagram of memory misdirection**. Here, the mechanism affected is undifferentiated “reasoning” (further distinctions might be made 1 day). Categories are based upon the various ways to control this.

Even if someone perceives and remembers the method used in a magic trick, this does not guarantee that it will be understood as contributing to the effect. Thus, magicians also manipulate the formation of your beliefs about what you just saw. In contrast to the last two categories (and perhaps reflecting our relative lack of knowledge about higher-level cognition), the misdirection of reasoning and beliefs is based on a set of techniques that are currently more loosely defined, and with a less-comprehensive structure (Figure 9).

#### Ruse

At the back of every spectator's mind lies the question as to *why* the magician carried out a particular action. For example, after seeing the magician make a coin disappear you might wonder why his hand went into his pocket: Was this the moment he got rid of that coin? A *ruse* is an action that misdirects the spectator's reasoning as to why an action was carried out. Magicians frequently use ruses to cover the true purpose of an action (Fitzkee, 1945; Lamont and Wiseman, 1999). The use of ruse is similar to the use of justified actions in perceptual misdirection [Section Internal (contextual) triggers], although applied to how people interpret the event rather than whether it has been registered in the first place.

#### Feigning actions

Experiencing magic requires people to not discover the true cause of the effects. One way of doing this is to have them make false attributions about the cause. As such, much of magic involves *feigning* actions whereby the magician pretends to do one thing when in fact he does something entirely different. In the French Drop for example, the magician pretends to transfer the coin from one hand to the other when in fact it remains in the original hand (Supplementary Video 1). Such methods only work as long as the spectator incorrectly interprets the event. Many different techniques can help magicians misdirect the way events are interpreted.

The *false transfer* is another commonly-used way of making small objects vanish. The magician pretends to hold a coin in his hands for several seconds before revealing an empty hand; this delay prevents people from suspecting a false transfer. Here the magician exploits the concept of object permanence, whereby we continue to perceive objects as present even when they are not directly visible. These forms of concealment also allow the magician to increase the delay between the method and the effect.

Several techniques can strengthen these effects; these are commonly known as *convincers*. For example, magicians may exploit cross-modal attribution errors to misdirect people toward believing that the object is still present. For example, in a coin vanish, the magician may use a false transfer which gives the impression that the coin has been transferred to the other hand. To further convince the audience that the coin is indeed in the other hand, he could produce a sound that convinces people that the coin is still in his hand by tapping the mimed coin on the table and generating the sound source through some other means (e.g., taping a real coin under the table) (Ganson, 1980).

#### Wrong assumptions

Each member of an audience has a set of pre-existing assumptions about the nature of the magic show, assumptions that they bring along to the performance. Whilst some of these assumptions are correct, others are not. Much of misdirection involves manipulating and exploiting these assumptions. These include the following principles:

***Dual reality***. Many magic tricks involve interactions between the magician and a selected member of the audience. There is an implicit assumption that the selected member experiences the same sequence of events as does the rest of the audience. But this assumption is often false. Consequently, magicians often exploit the misalignment between different people's understanding of an event, known as the principle of *Dual Reality*. For example, the magician might use trickery to ensure the volunteer experiences a different event compared to the rest of the audience, while using linguistic subtleties to convince both parties that they experienced the same events. The concept of dual reality is an extremely powerful principle in magic.

***Multiple outs***. Most people assume that a magic trick has a single pre-determined end. However, many tricks have multiple possible endings, allowing the magician to choose between them, depending on what other choices have been made. For example, multiple predictions for each of the numbers 1–4 could be in an envelope; the magician would remove only the appropriate one based on the spectators choice. The principle of multiple outs is a powerful method that uses linguistic cues to misdirect people's interpretation of the event. Moreover, it also relies on peoples' erroneous assumptions about the nature of magic tricks (i.e., all tricks have a pre-determined end).

***Effort put into an effect***. It is difficult for non-magicians to realize how much time, effort and money can be put into what might appear to be a simple trick (Teller, 2012). Thus, people will often exclude potential solutions to a trick simply because they believe that no-one would go to so much effort just to create it. This false assumption is powerfully exploited when magicians pretend to perform a trick as an impromptu demonstration (whereas in reality vast amounts of preparation have gone into preparing it). This might explain why people tend to experience impromptu magic demonstrations as being more impressive than large-scale stage illusions.

***Pre-show***. Another false assumption commonly made is that magic tricks begin when the performer says they begin. However, many magicians use pre-show work to gather information about members of the audience, which can then be used later on in the show. The misdirection here involves using subtle forms of language and deception that prevent the other audience members from realizing that this information could have been gathered beforehand.

## Conclusion

Performing magic does not necessarily require a deep understanding of why misdirection works; most magic practitioners are simply interested in improving their magic performance. Consequently, previous taxonomies of misdirection have tended to emphasize those aspects dealing directly with technique.

However, in recent years there has been increased interest in understanding why these techniques (and their related principles) work, ideally by linking them to what is known of human cognition (Kuhn et al., 2008a). To facilitate this, we have proposed here a way to organize knowledge about magic (or at least, misdirection) such that is based on our current understanding of perception and cognition. Our psychologically-based taxonomy is far from complete, and as our understanding of both misdirection and cognition advance, aspects of this taxonomy will change. But we envisage that it will help the dialog between magicians and scientists and act as a useful perspective from which to explain the psychological mechanisms involved. Among other things, we hope that it will help highlight misdirection principles to an audience with less knowledge in magic. We also hope that it might provide a template for a similar organization of knowledge about other aspects of magic more generally (see also Rensink and Kuhn, under review).

Defining misdirection has been far from trivial, and there is still no general consensus on its definition. We chose a rather broad definition of misdirection so as to include a wide range of cognitive mechanisms. If our definition is too broad, we could be in danger of developing a taxonomy of magic in general rather than misdirection. Whilst Hugard (1960), implicitly suggests that misdirection and magic can indeed be used synonymously, we do not intend to develop a complete taxonomy of magic here. Indeed there are countless magic principles that do not fall within our taxonomy, in that they do not involve misdirection (e.g. forcing, optical illusions, suggestions…).

Magicians are undoubtedly masters of deception. But they tend to be skeptical about whether science can teach them anything about misdirection, or magic in general (Teller, 2012). In most other domains (e.g., medicine or sports), practitioners have improved performance by understanding the mechanisms involved. It's hard to see why magic should be an exception. Thus, although our psychologically-based taxonomy is primarily intended to further our understanding of cognition, it may well help magicians improve their misdirection. To begin with, it could help magicians draw links between misdirection and formal theories of cognition, which could help them develop more effective tricks. For example, there is much scientific knowledge about several rather counter-intuitive cognitive biases and illusions (e.g., change blindness, inattentional blindness, false memories, choice blindness), which helps explain the mechanisms behind these illusions. And as in any other domain, it is likely that knowledge about the cognitive processes will eventually lead to improvements in the methods used, and maybe even new misdirection principles (see also Williams and McOwan, 2014; Rensink and Kuhn, under review). In any event, we hope that our taxonomy will encourage further scientific research in the field, and so help us better understand the human mind.

### Conflict of interest statement

The authors declare that the research was conducted in the absence of any commercial or financial relationships that could be construed as a potential conflict of interest.

## Footnotes

^1^ Throughout the manuscript we refer the reader to videos that describe some of the misdirection methods (see supplementary material).

^2^ Interestingly, the kinds of subjective experience created by Ascanio's three grades of misdirection appear to loosely correspond to the three grades of visual experience posited as resulting from different levels (or kinds) of attention (Rensink, 2013, 2015).

^3^ Interestingly, the active-passive distinction corresponds somewhat to the two forms of attentional control believed to exist by vision scientists: *exogenous* control (reflexive control based on events such as a sudden change in movement or sound), and *endogenous* control (higher-level, conscious control based on the observer's understanding of a situation). However, endogenous control can be based on dynamic as well as static stimuli, something contrary to Sharpe's characterization.

^4^ As in the case of Sharpe (1988), the physical-psychological distinction corresponds somewhat to the exogenous- endogenous distinction generally made by vision scientists. However, endogenous control of attention can involve any aspect of conscious cognition, and not just suspicion.

^5^ Although such a taxonomy would be stable for the most part, it might change slightly on occasion to incorporate the latest discoveries about psychological mechanisms. Conversely, it might also help determine these.

^6^ Although these systems are fairly distinct, there is still some degree of overlap. For example, memory of a kind exists in all perceptual processes (e.g., iconic memory in visual perception). Some forms of reasoning also take place at a perceptual level (in that they need some intelligence to interpret the incoming signals). However, these can be readily distinguished from their higher-level equivalents in several ways. Perhaps most importantly, they are much less flexible, and so much less prone to being manipulated. For example, the contents of any visual memory simply reflect what has been processed by the visual system—it cannot have contents that differ from this. Likewise, any assumption used by perceptual processes (e.g., that lighting comes from above) cannot be altered; it can only be overridden by higher-level control. Practically speaking, then, the division proposed here is a reasonable one for present purposes.

^7^ Lamont and Wiseman also treat attention and eye movements synonymously even though (as mentioned earlier) the two can be dissociated.
