# Supplementary material for: A psychologically-based taxonomy of misdirection
Source: Front Psychol. 2014 Dec 9;5:1392. doi: 10.3389/fpsyg.2014.01392 (PMC4260479; doi:10.3389/fpsyg.2014.01392)
Supplement: Supplementary file 5 [file DataSheet1.DOCX]

A: French drop vanish

The magician displays a coin in his hand. He takes it in his other hand to vanish it by rubbing it away. Eye gaze and posture are used to focus the viewer's attention on the “vanishing” hand (technique 2.1.1.1-ii). The viewer's reasoning about what has actually occurred is also influenced by taking advantage of object permanence by feigning the action of taking the coin (technique 2.3.2). The magician waits for a moment before revealing that the coin has vanished, using a delay to separate the true cause from the effect (technique 2.2.1). The false transfer is done without fanfare, whereas the moment of the vanish involves a pause and a meaningful movement of the hand, manipulating attentional focus by implicitly suggesting that nothing important happens at the time of the method (technique 2.1.1.1.2 and 2.1.1.2-ii). These principles are common to many effects that use sleight of hand.

B: pen vanish

The magician displays a coin in his hand. He taps it with a pen (using it like a wand), when suddenly it vanishes. He reveals that he simply placed the pen in his collar and retrieves it – but when he goes to tap the coin again, this time the coin has vanished. Attentional misdirection techniques used include physical and social cues to control focus (techniques all used in supplementary video A), as well as timing & repetition to reduce interest in the pen when it vanishes on the third tap (technique 2.1.1.1.2-iii). The surprise about the vanish and location of the wand is also used to cover the subsequent vanish of the coin (technique 2.1.1.1.2-ii), which happens while being physically masked (technique 2.1.2-i). Both effects in this trick happen “outside of the effect” as well: the pen vanish is unexpected, and the coin vanish is done after the trick appears to be over (technique 2.1.1.2-i).

C: Eating a lighter

The magician holds a lighter in his hands. After a warning to not try this trick at home, he apparently eats the lighter, and his hands are empty. The lighter is dropped into the magician's lap during an off-beat created before the effect, using misdirection to control the timing of attention (technique 2.1.1.2-ii).

D: Cut prediction

The magician sits at a table with a deck of cards, their box, and a large card visible underneath the box. He invites a spectator to cut the cards, explains that he has made a prediction about the spectator's actions, and asks for the spectator's chosen card to be shown to the camera. He then reveals that the prediction accurately matches the seemingly randomly-selected card. The misdirection in this trick relies on the spectator forgetting which card they cut to, with the magician providing a misleading recap during a delay to facilitate a false memory that will also impact reasoning about the effect (techniques 2.2.1 and 2.2.2).
